# AISO: Annotation of Image Segments with Ontologies

**DOI:** 10.1186/2041-1480-5-50

**Published:** 2014-12-17

**Authors:** Nikhil Tej Lingutla, Justin Preece, Sinisa Todorovic, Laurel Cooper, Laura Moore, Pankaj Jaiswal

**Affiliations:** School of Electrical Engineering and Computer Science, Kelley Engineering Center, Oregon State University, Corvallis, OR 97331-2902 USA; Department of Botany and Plant Pathology, Oregon State University, 2082 Cordley Hall, Corvallis, OR 97331-2902 USA

**Keywords:** Image annotation, Semantic web, Plant ontology, Image segmentation, Plant anatomy, Web services, Computer vision, Image curation, Machine learning

## Abstract

**Background:**

Large quantities of digital images are now generated for biological collections, including those developed in projects premised on the high-throughput screening of genome-phenome experiments. These images often carry annotations on taxonomy and observable features, such as anatomical structures and phenotype variations often recorded in response to the environmental factors under which the organisms were sampled. At present, most of these annotations are described in free text, may involve limited use of non-standard vocabularies, and rarely specify precise coordinates of features on the image plane such that a computer vision algorithm could identify, extract and annotate them. Therefore, researchers and curators need a tool that can identify and demarcate features in an image plane and allow their annotation with semantically contextual ontology terms. Such a tool would generate data useful for inter and intra-specific comparison and encourage the integration of curation standards. In the future, quality annotated image segments may provide training data sets for developing machine learning applications for automated image annotation.

**Results:**

We developed a novel image segmentation and annotation software application, “Annotation of Image Segments with Ontologies” (AISO). The tool enables researchers and curators to delineate portions of an image into multiple highlighted segments and annotate them with an ontology-based controlled vocabulary. AISO is a freely available Java-based desktop application and runs on multiple platforms. It can be downloaded at http://www.plantontology.org/software/AISO.

**Conclusions:**

AISO enables curators and researchers to annotate digital images with ontology terms in a manner which ensures the future computational value of the annotated images. We foresee uses for such data-encoded image annotations in biological data mining, machine learning, predictive annotation, semantic inference, and comparative analyses.

## Background

Annotation of Image Segments with Ontologies (AISO) is an interactive tool which allows users to segment and annotate a digital image – such as those produced with digital photography or from scanned prints – with ontology terms. An ontology is a controlled and structured vocabulary of agreed-upon labels (‘terms’) that represent the knowledge of the types of entities within a given domain [1]. Labeling image data with ontology terms imbues it with semantic meaning, which makes it possible to computationally infer relationships amongst different images and parts of images. The use of ontologies has gained increasing importance as the number, complexity, and size of biological data sets have increased [2]. AISO was developed in response to a need within the biology community for a streamlined tool that enables consistent and structured labeling of digital images. A shift in research focus towards high throughput phenotyping [3, 4] requires specialized tools that bring consistency to the image annotation process. AISO annotates images with ontology terms and taxonomy labels via lightweight web services, allowing users to select and annotate image segments.

Many photo-editing and illustration software packages enable the *ad hoc* editing of an image, but any highlighting and labeling utility requires thorough knowledge of the software’s illustration capabilities (i.e. layering, boundary detection) and does not include the structured integration of scientific data. For example, any labels applied to hand-illustrated segments superimposed onto an image would have to be individually constructed and associated with a particular portion of an image. AISO simplifies this functionality and requires only a few input gestures and clicks to identify and label segments. The resulting structured image and ontology annotation allows for consistent extraction techniques, enabling future database storage, active learning, and semantic inference functionalities. Researchers are thus empowered to construct meaningful image data sets drawn from their laboratories, online image archives, and publications.

## Implementation

### Software architecture

AISO is a multi-platform, Java desktop application extending the source code of the Interactive Segmentation Tool (IST) [5], originally developed for comparing the performance of image segmentation algorithms. The user interface was constructed using the Standard Widget Toolkit [6], an open-source Java package. The ontology terms are provided through a light-weight Plant Ontology web service [1], and returns data in the JSON format [7]. Species names are provided via the uBio web service [8] in XML. Annotation data -- segments, labels, and curation details -- are all saved into a compressed ZIP archive, which contains the original image, binary segment data files, segment mask images, and an XML file storing segment coordinates and other curation metadata. An example of the contents and structure of an AISO XML metadata file is available in the Help document, which may be accessed from the application menu.

### Choice of segmentation algorithm

Segmentation algorithms are created with different application domains in mind and the computer vision research community is generally focused on segmenting images of the human body and the built environment. Segmenting anatomical images of biological specimens, such as plants, presents a number of challenges that have received scant attention in the literature. Plants contain curvilinear and asymmetric forms, textures, and spatial orientations that make identification and classification more difficult for computer vision algorithms. We chose the Interactive Graph Cuts (IGC) segmentation algorithm because markups have a local effect, thereby avoiding major global deformations in the segmented area. This has great value in plant images that contain densely grouped features, such as many similar, overlapping leaf structures. The IGC algorithm also is more accurate in extracting foreground objects, and includes a responsiveness that allows the user to iteratively refine the segments. The average time required for a user to attain optimal object and boundary accuracy for an image, and the average total time spent annotating each image are much lower when compared to other algorithms [9].

## Results and discussion

### Segmenting and annotating images

**Figure 1:**
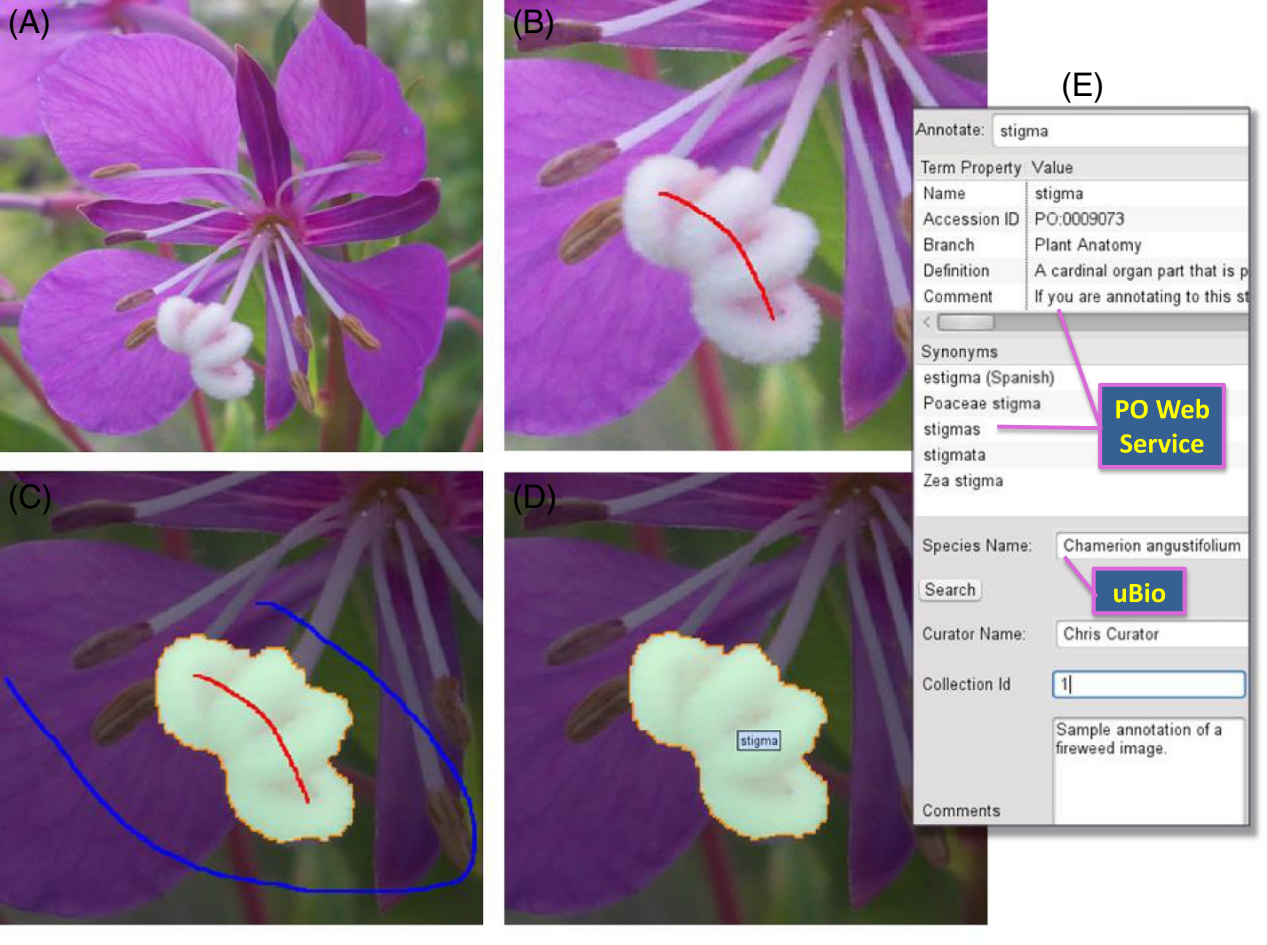
**Screenshots of AISO demonstrating the segmentation process. (A)** Open an image file in AISO (*e.g.* a *Chamerion angustifolium* flower) **(B)** Mark the “foreground” (red line): the aspect of the image you would like to segment. **(C)** Mark the “background” (blue line): the area of the image you want to ignore. (NOTE: Auto-segmentation occurs after the background is marked; further refinement of both foreground and background is possible.) **(D)** Label the new image segment with an ontology term selected from the integrated web service query interface. **(E)** Screenshot of the annotation panel of AISO showing a selected ontology term, its definition and synonyms, and designated species (provided via web services), as well as curator, collection, and comment information entered by the user.

After opening an image file in AISO (Figure 1A; allowed formats: JPEG, PNG, GIF, and BMP), the user works in two alternating modes: Segmentation and Labeling. The default Segmentation mode operates in a foreground/background paradigm and allows the user to mark the “foreground” of an image by drawing red lines with the mouse or trackpad (left-click and drag); the “background” is denoted with blue lines (right-click and drag). Once both the foreground and the background are designated with these “mark-up” lines (Figure 1B,C), AISO will execute its IGC segmentation algorithm [10], identifying and extracting borders circumscribing the foreground mark-up and visually highlighting the area (Figure 1C). Edges in an image can be extracted wherever there is a detectable change in the lightness or darkness of adjacent pixels. The user may further refine their segmented area by adding foreground mark-up lines (red) outside of the initial area to expand the discernable boundary, or by applying background mark-up lines (blue) across the initial area to exclude nearby image content from the segment. Once satisfied with a particular segment, the user must form that segment by pressing the “Form segment” button, thereby fixing it as a new layer overlaid on top of the original image. Following segmentation, the user then enters into Labeling mode by pressing the “Labeling Mode” button to assign an ontology term to a segment. The user may search for a Plant Ontology term by typing its name in the dropdown box labeled “Annotate”. The PO terms are provided through a web service [1] which requires an Internet connection. The dropdown box will display auto-completed term suggestions when the user presses the down arrow on their keyboard. After selecting a term in the dropdown, the user should press the “Assign” button to associate their selected PO term with the currently selected segment. The assigned ontology term will appear on a segment when the user selects that segment and hovers over it with the pointer (Figure 1D). The user may also assign a taxonomic name to the entire annotated image via the uBio namebank search web service [8] and enter additional curation metadata (Figure 1E).

The user can save annotated image files into a custom ZIP package, and may also re-open previously annotated images for continued editing. The original image is always preserved and viewable. When saving annotations, the user may optionally export an HTML file containing a web-enabled version of the annotated image, which allows the user to easily share their work in other media platforms. For example, manuscript authors could submit annotated images along with other supplementary data, to enhance the collection of ontology-based image data for comparative analyses and machine learning. Annotated images could thereafter be used in online resources and publications, or placed in a file archive or database for future analysis.

### Case studies

**Figure 2:**
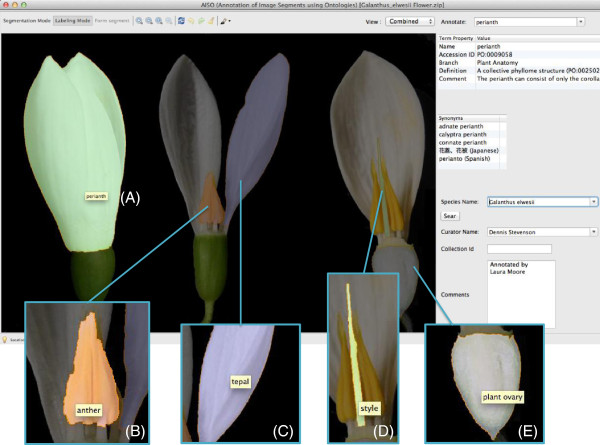
**Screenshots and insets of AISO displaying annotated segments of** ***Galanthus elwesii*** **(giant snowdrop) flowers.** The represented segments are **(A)** *perianth* (PO:0009058), **(B)** multiple instances of *anther* (PO:0009066), **(C)** *tepal* (PO:0009033), **(D)** *style* (PO:0009074) and **(E)** *plant ovary* (PO:0009072).

AISO was used to segment and label small library of botanical images (provided by Dennis Wm. Stevenson; New York Botanical Gardens), both as a test of AISO’s capabilities and as the beginnings of a training set of segmented image data for a potential future active-learning system. One such image of the floral structures of *Galanthus elwesii*, commonly known as “Giant Snowdrop”, was segmented and labeled with the Plant Ontology terms *plant ovary* (PO:0009072), *style* (PO:0009074), *tepal* (PO:0009033), *anther* (PO:0009066) and *perianth* (PO:0009058) (Figure 2). Each segment was generated with only a few user-directed mouse or trackpad strokes, followed by a quick auto-complete search for the appropriate Plant Ontology term. Each segment’s overlay is assigned a different preset color to help distinguish it from any other segments in the same image.

**Figure 3:**
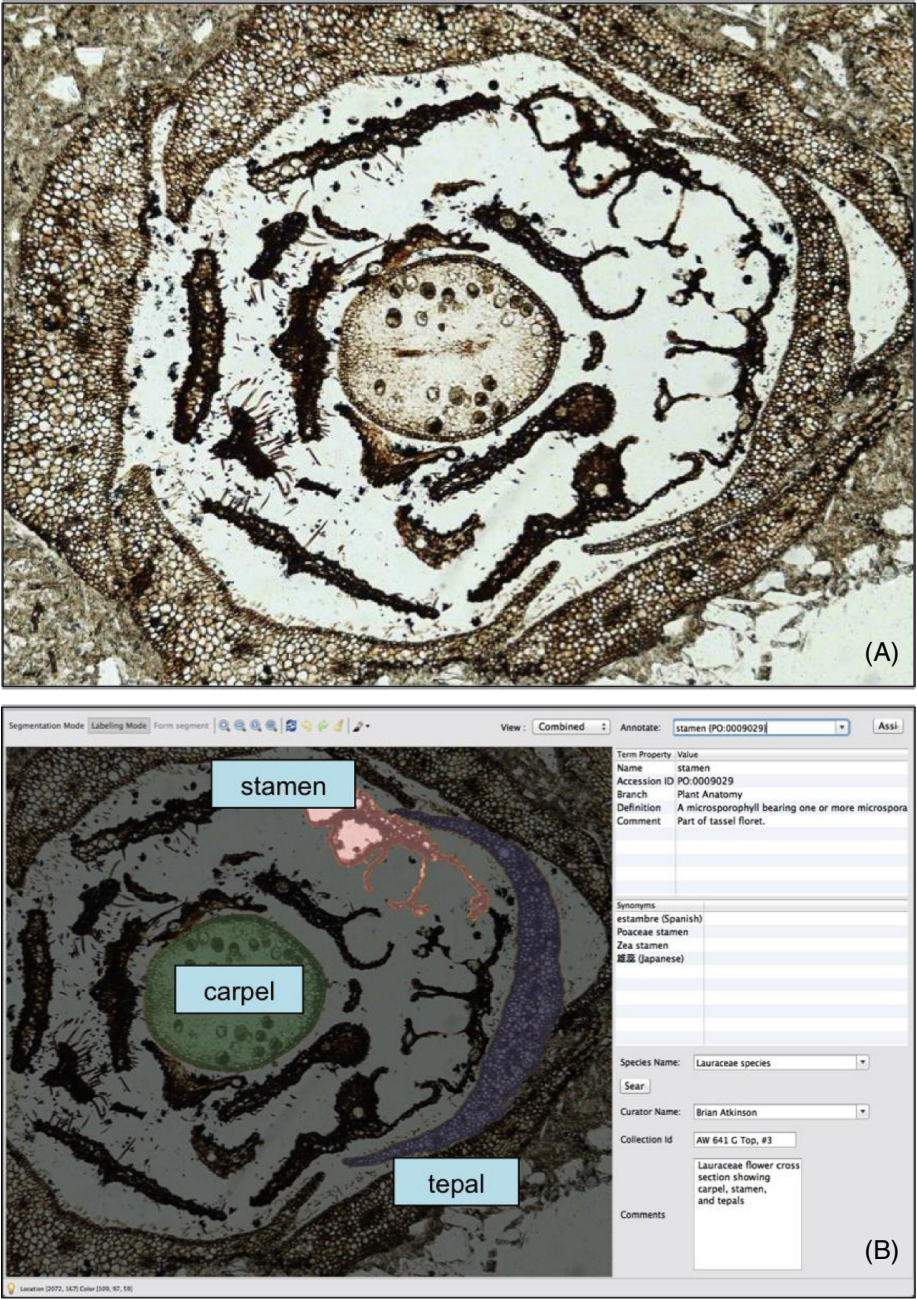
**Cross section of a fossilized lauraceous flower from the Eocene epoch (~55 MA) showing a single carpel, stamen, and tepals.** The original image **(A)** has been segmented and annotated using Plant Ontology terms in AISO **(B)**. Note the automated color differentiation between segments. The annotation labels in this figure have been enlarged for readability.

Additionally, AISO’s capabilities have been applied to paleo-botanical images of fossilized lauraceous flowers from the Eocene epoch (Figure 3). In this particular case, Plant Ontology terms *stamen* (PO:0009029), *carpel* (PO:0009030), and *tepal* (PO:0009033) were applied to user-identified segments in a cross-section image. This particular example highlights the IGC algorithm’s localized border-detection capabilities when constructing an image segment.

### Comparison to existing software

AISO brings together disparate image segmentation and semantic labeling functionalities found in existing software and merges them into a user-friendly, science-focused package. Hollink *et al.* [11] developed an application interface for annotating whole images with ontology terms, but it lacked an image segmentation feature. Conversely, Shao *et al.* [12] developed image segmentation capabilities without segment-specific semantic tagging features. Semantic Image Annotator [13], built as an extension to the web-focused Semantic MediaWiki platform, allows users to define rectangular areas on an image file and tag those areas with semantic labels, but does not provide dynamic image segmentation. Koletsis and Petrakis’ dissertation work [14] includes an algorithm named “Semantic Image Annotation” which automatically annotates images with ontology terms based on a training data set of similar images, but this approach also lacks a segmentation feature.

### Future development

Future enhancements to AISO include extending web service support for multiple ontologies, such as those developed by OBO Foundry [15] members and model organism databases. Enhancements would also include enabling automated segmentation based on active learning, and adding support for high-resolution images (10–120 megabytes).

## Conclusions

AISO allows researchers and curators to interactively segment images and assign semantic annotations to those segments. This annotation capability gives biologists the opportunity to enhance the computational value of their own image data. Data-enriched images can be used to mine biological data sets, train machine learning software, and generate conclusions via semantic inference. We believe that the existing functionality of AISO, combined with our future efforts in active learning, will provide a powerful tool for the biology community and for scientific journals interested adding annotated images and associated metadata to their publication pipeline.

### Availability and requirements

**Project name:** Annotation of Image Segments with Ontologies (AISO).

**Project home page:**http://www.plantontology.org/software/AISO.

**Operating system(s):** Platform-independent (Mac OS X, Linux, Windows).

**Programming language:** Java.

**Other requirements:** An Internet connection, the Java Runtime Environment (JRE).

**License:** Creative Commons (Attribution-NonCommercial-NoDerivs 3.0 Unported).

**Any restrictions to use by non-academics:** No.

## Abbreviations

AISO: Annotation of image segments with ontologies
IGC: Interactive graph cuts
IST: Interactive segmentation tool
JSON: Javascript object notation
PO: Plant ontology
XML: Extensible markup language.
